# Transient process spectroscopy for the direct observation of inter-molecular photo-dissociation

**DOI:** 10.1063/1.4983639

**Published:** 2017-05-16

**Authors:** Sena Hashimoto, Atsushi Yabushita, Izumi Iwakura

**Affiliations:** 1 Department of Applied Chemistry, Graduate School of Engineering, Kanagawa University, 3-27-1 Rokkakubashi, Yokohama 221-8686, Japan; 2 Research Institute of Engineering, Kanagawa University, 3-27-1 Rokkakubashi, Yokohama 221-8686, Japan; 3 Department of Electrophysics, National Chiao-Tung University, Hsinchu 300, Taiwan; 4 Department of Chemistry, Faculty of Engineering, Kanagawa University, 3-27-1 Rokkakubashi, Yokohama 221-8686, Japan

## Abstract

Transient process spectroscopy has previously been thought to be applicable only to the analysis of intra-molecular processes. Two metal ion bridges used in the present work have allowed us to visualize real-time variations of the molecular vibration frequencies during photo-disproportionation inside bimolecule aggregates, which directly shows transient inter-molecular reactions.

## INTRODUCTION

**FIG. 1.**
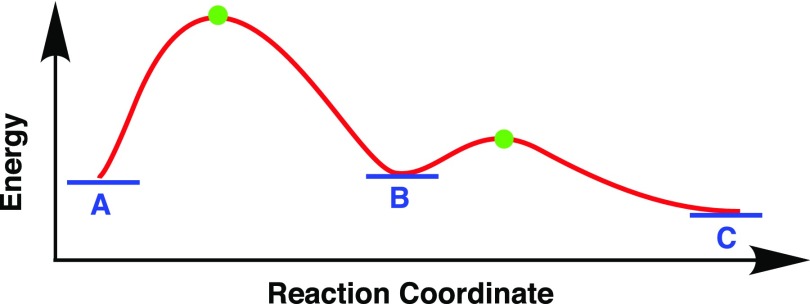
Reaction pathway (A → B → C) and transient processes (red curve) for a typical chemical reaction. A is the reactant, B is an intermediate, C is the product, and the green dots indicate transition states.

Rapid processes that cannot be tracked visually can often be observed by acquiring photographic images under rapid strobe lighting. The development of femtosecond strobe lights has enabled the study of the electronic and vibrational dynamics of transition states in photo-reactions. As a result, reaction pathways such as A → B → C in Fig. 1 can now be observed.^1^ The availability of 5-fs laser pulses,^2^ the duration of which is much shorter than molecular vibration periods, has enabled real-time observations of atomic motions as well.^3^ The burst capture of strobe spectra has also allowed the tracking of transient processes such as bond breaking and bond reformation (indicated by the red curve in Fig. 1) in chemical reactions.^4^

The functional groups of organic compounds typically generate specific absorption bands in the range of 1000–2000 cm^−1^, corresponding to their molecular vibrational modes. As an example, the stretching modes of C=C and C-C bonds appear at 1600 and 1100 cm^−1^, in association with vibrational periods of 20 and 30 fs. These molecular vibrations can be temporally resolved by measurements employing sub-10 fs laser pulses that allow observations of vibrational motions in real-time. In addition, examination of changes in instantaneous molecular frequencies allows the visualization of transient processes such as chemical bond breaking and reformation. The observation of transition states has also been reported in the case of various intra-molecular reactions, based on the use of sub-10 fs laser pulses.^4–6^

The majority of organic photo-reactions proceed as inter-molecular reactions via inter-molecular collisions or inter-system crossing occurring in the picosecond to nanosecond time scale. However, the coherent molecular vibrations produced by impulsive photoexcitation dephase as fast as a few picoseconds,^5,7^ and moreover, the inter-molecular collision destroys the coherence of the molecular vibration. Therefore, previously it had been thought to be impossible to observe the inter-molecular reactions via coherent molecular vibration dynamics. The present work investigated a compound consisting of two molecules (anion dimer) bridged by two metal ions. The bridged structure allowed us to observe coherent molecular vibration dynamics suppressing inter-molecular collision.

## EXPERIMENT

### Ultrashort visible pulses

A Ti:sapphire regenerative amplifier (SpectraPhysics, Spitfire model) was used to generate near infrared (NIR) femtosecond pulses (duration 100 fs, central wavelength 800 nm, repetition rate 1 kHz, and pulse energy 3 mJ) so as to produce ultrashort visible pulses using a home-made non-collinear optical parametric amplifier (NOPA).^8^ The setup of the optical system is almost the same as the one previously described in detail.^16^ In the previous work, we have compressed the pulse duration using a pulse compressor consisting of a diffraction grating and a deformable mirror; however, high order chirp was still remaining in the compressed pulse. Thus, we have added a chirped mirror pair to compensate the high order chirp component. The amplified broadband visible pulse, extending from 500 to 740 nm, was compressed to a sub-10 fs pulse.

### Real-time measurements of vibrational motions in molecules

A typical molecular vibration has a period of 20 fs, and so cannot be temporally resolved by time-resolved spectroscopy systems employing laser pulses with durations of 35 fs or longer. Therefore, ultrashort visible laser pulses with sub-10 fs durations have been developed, since these can temporally resolve real-time molecular vibrations. To allow the observation of both electronic and vibrational dynamics, a time-resolved absorption pump-probe spectroscopy system was designed in the present work, as follows.

In this system, the intense broadband visible laser pulse generated by the NOPA was separated into two copies, at a power ratio of 10:1, and these were employed as the pump and probe pulses in pump-probe spectroscopy. The chirps of the pump and probe pulses were adjusted to have a pulse duration of less than 10 fs at the point of impingement on the solution sample held in a synthetic fused silica glass cell. The chirp adjustment was accomplished as follows.

The glass cell used in the present work (GL Sciences Inc., S15-IR-1) has 1-mm optical path length and its glass walls have a thickness of 1.1 mm. We have broken one of the glass cells into half to get one of the glass walls of the glass cell. The glass wall plate was inserted in front of the beam sampler in the pump-probe setup. Thus, both the pump pulse and the probe pulse transmit through the glass wall plate for once. By using a parabolic mirror with 101.6-mm reflected focal length, these pulses were focused in the beta barium borate (BBO) crystal with 10-*μ*m thickness to generate the sum frequency between the two pulses. The sum frequency pulse was clipped by an iris and coupled into optical spectral analyzer. The sum frequency spectrum was measured as a function of the delay between the pump and probe pulse in the second harmonic generation (SHG) frequency resolved optical gating (FROG) measurement. Therefore, the pulse duration estimated in the SHG FROG measurement reflects that of the pulse in the glass cell for the solution sample. We have adjusted the grating compressor to let the estimated pulse duration as short as possible in this situation. Thus, the visible pulse transmitted through one glass wall plate has a pulse duration of sub-10 fs. On the measurement of the solution sample, we have removed the glass wall plate and put the solution sample filled in the glass cell. Therefore, the solution sample was excited and probed by the sub-10-fs ultrashort visible pulse.

The probe pulse transmitted through the sample was coupled into a polychromator (Princeton Instruments, SpectraPro 2150i). The probe spectrum was subsequently dispersed by the polychromator and measured by a fast scan rate CCD line scan camera (Entwicklungsbuero Stresing, Series 2000) with a line scan rate of 1 kHz. The probe spectrum was acquired for every pulse at a repetition rate of 1 kHz. The pump pulse was modulated by a mechanical chopper running at a frequency equal to half the laser repetition rate. Thus, the acquired probe spectrum contained the signal from the sample excited by the pump pulse (*T* + Δ*T*) and the signal from the sample in the unexcited state (*T*) for every two probe pulses. Absorption changes were calculated as ΔA = −log10 (1 + Δ*T*/*T*). The absorption changes were determined by scanning the optical delay between the pump and probe pulses at 500 ms intervals with a step-size of 0.2 fs, from −30 to 1800 fs. In the subsequent data analysis, we averaged every five delay points to improve the signal-to-noise ratio.

### Sample

A saturated methanol solution of 2,2′-(2,5-Cyclohexadiene-1,4-diylidene) dimalononitrile (TCNQ)^9^ was stored in a glass bottle and placed under natural light for five weeks to produce a radical anion dimer bridged by two sodium cation (Na^+^_2_[TCNQ^−•^]_2_). A transition from the original yellow color of the initial TCNQ solution to green provided evidence for the charge transfer (CT) band of the [TCNQ^−•^]_2_ at 643 nm.^10–13^ A portion of the resulting Na^+^_2_[TCNQ^−•^]_2_ solution, with a concentration of 3.6 × 10^−4^ M, was transferred into a fused silica cell in preparation for the pump-probe measurements. The glass cell was put on a mechanical motorized stage to be continuously moved in a circle shape in a plane orthogonal to the light path. Thus, the system has been adjusted to implement that the sample probed by the last pulse will not be probed by the next pulse coming 1 ms later. The sample existing at the irradiated point goes away by convection in the glass cell after running the glass cell through the whole arc of the circle. Therefore, the degradation effect of the sample can be excluded during the measurement. It was also confirmed that the stationary absorption spectrum of the sample does not show any recognizable difference between before and after the measurement. All trials were performed at a room temperature of 22 ± 1 °C.

### Theoretical calculations

The Gaussian 09 program^14^ was used for calculations, without assuming symmetry. Theoretical calculation for Na^+^_2_[TCNQ^−•^]_2_ was performed replacing sodium with potassium. Geometric optimization was performed at the B3LYP/6–31G* level, and 5*d* functions were employed for the *d* orbitals. Raman active molecular vibration frequencies were calculated for each of the obtained structures at the same level.

## RESULTS AND DISCUSSION

**FIG. 2.**
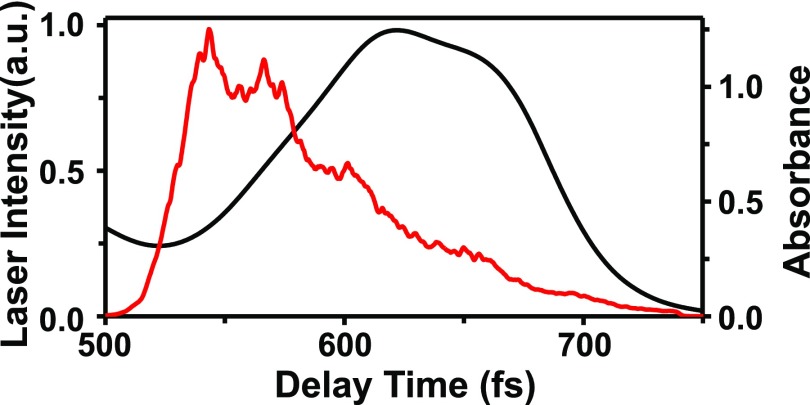
Spectrum of the ultrashort visible laser pulse (red curve) and the absorption spectrum of the methanol solution of Na^+^_2_[TCNQ^−•^]_2_ (black curve).

The spectrum of the ultrashort visible laser pulses overlapped most part of the CT absorption band of the methanol solution of Na^+^_2_[TCNQ^−•^]_2_ (Fig. 2). Therefore, these pulses were able to trigger the photo-disproportion reaction: [TCNQ^−•^]_2_ → TCNQ + TCNQ^2−^.^12,15^ It results in that the electronic decay dynamics observed in the vicinity of 700 nm (see Appendix A) reflects the stimulated emissions of [TCNQ^−•^]_2_ and TCNQ^2−^.^16^ The former exhibited decay within the observed delay region, while the latter showed a rise and subsequent decay. We have studied this reaction process analysing molecular vibration dynamics of the transient absorption trace probed at 700 nm as follows. The observed dynamics was confirmed performing the same analysis also at other probe wavelengths (see Appendix B).

**FIG. 3.**
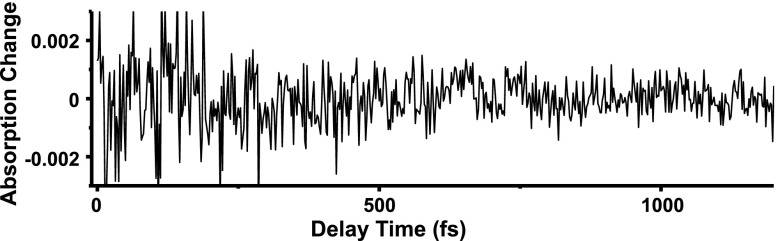
Oscillating components of the absorption change trace probed at 700 nm.

The transient absorption trace includes slow relaxation reflecting electronic dynamics and fast oscillation reflecting vibrational dynamics. We have an applied high pass filter to the transient absorption trace and got the fast oscillating components, which is plotted in Fig. 3. The fast oscillation of the trace was caused by the wave packet motion on the potential energy surface oscillating with the period of the molecular vibration. The molecular vibration dynamics can be examined via a spectrogram analysis.^17^ Scalogram analysis (see Appendix C) was also performed to confirm the vibrational dynamics observed by the spectrogram analysis. The spectrogram is obtained by short-time Fourier transform (STFT).^18,19^ Calculation of the spectrogram is performed to obtain Fourier transform of the product of the transient absorption trace ΔA(*t*) and the gate function g(*t − τ*) shifting *τ* (the center position of the gate function). When *τ* = *τ_0_*, the product ΔA(*t*)g(*t − τ*) contains oscillating components around *t* = *τ_0_* because g(*t − τ_0_*) is non-zero only in −T/2 < t − *τ_0_* < Τ/2. Therefore, its Fourier spectrum, i.e., spectrogram for *τ* = *τ_0_*, S(*ω*, *τ_0_*), represents the vibrational spectrum at around *t* = *τ_0_*. Calculating the Fourier transform shifting *τ*, spectrogram is obtained reflecting the dynamics of the molecular vibrational spectrum. The spectrogram was calculated using the Blackman window function with a full width at half-maximum of 200 fs, as shown in the below equation

$$\begin{array}{c} S(\omega ,\tau )=\int_{0}^{\infty }\Delta A(t)g(t-\tau )\text{exp}(-i\omega t)dt,\\ g(t)=\{\begin{array}{c} 0\left(t<-\frac{T}{2}\text{or}\frac{T}{2}<t\right)\\ 0.42+0.5\;\;\text{cos}\;\frac{2\pi t}{T}+0.08\;\;\text{cos}\;\frac{4\pi t}{T}(-\frac{T}{2}<t<\frac{T}{2}). \end{array} \end{array}$$ (1)

**FIG. 4.**
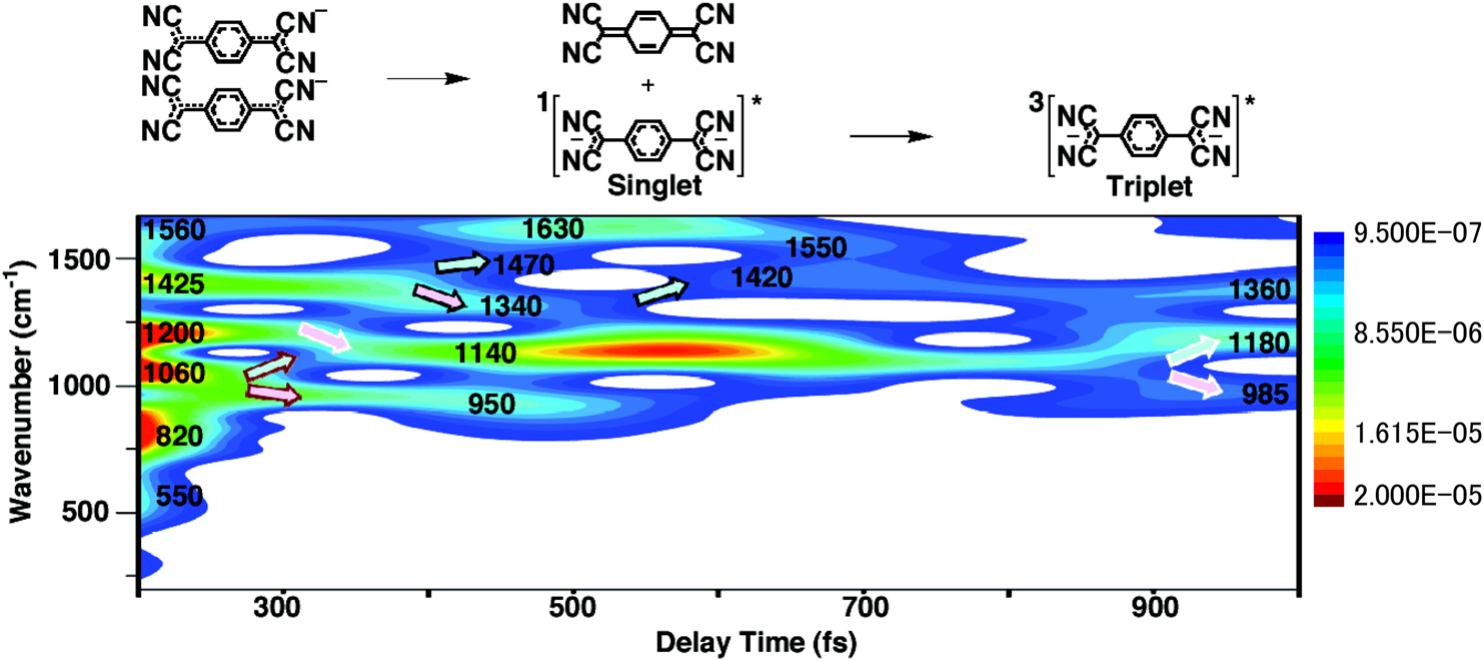
Spectrogram calculated from the measured absorption change trace.

The resulting spectrogram is provided in Fig. 4, where the horizontal axis, vertical axis, and color bar represent the delay time, the instantaneous molecular vibration frequency, and the signal intensity, respectively. The spectrogram was shown from 200 fs because the spectrogram is noisy at earlier than 200 fs reflecting the coherent artefact existing around the zero delay region. Density-functional theory (DFT) calculations at the B3LYP/6-31G* level were performed to assign the vibrational modes appearing in the spectrogram (Table I).

**TABLE I.** Molecular vibration frequencies predicted from theoretical calculation (cm^−1^). Values within parentheses are the ones resolved from the experiment.

	K_2_[TCNQ]_2_	TCNQ	TCNQ^2−^(singlet)	TCNQ^2−^(triplet)
$\nu_{\text{CCr}}$	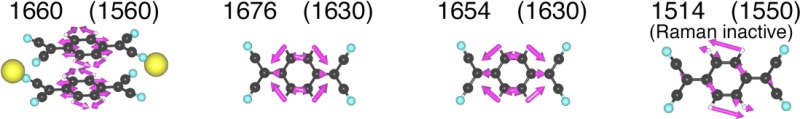			
$\nu_{\text{CCs}}$	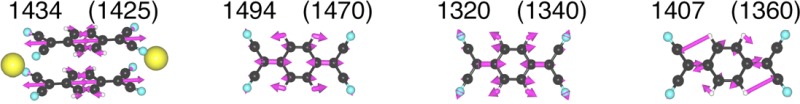			
$\delta_{\text{CH}}$	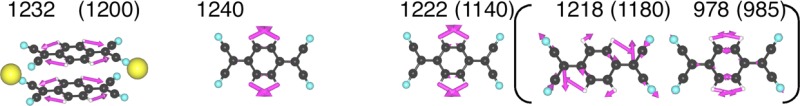			
$\nu_{\text{Bn}}$	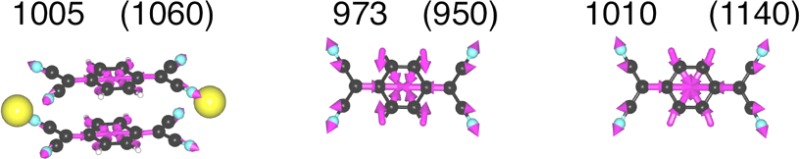			
$\delta_{\text{TCNQ}-\text{TCNQ}}$	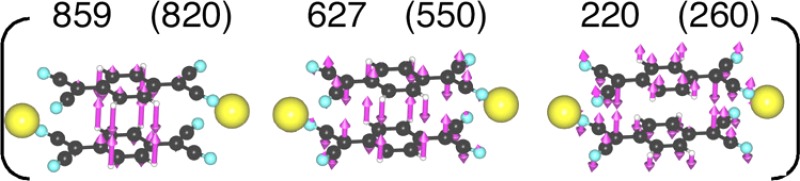			

The vibrational modes immediately after photo-excitation reflect those of Na^+^_2_[TCNQ^−•^]_2_. The frequency region below 820 cm^−1^ shows inter-fragment vibrational modes^20^ (*δ*_TCNQ-TCNQ_), while the peaks at 1060, 1200, 1425, and 1560 cm^−1^ are assigned to the symmetric stretching mode of the benzene ring (*ν*_Bn_), the C-H bending mode (*δ*_CH_), the C-C 1.5 bond stretching mode of the side chain group (*ν*_CCs1.5_), and the C-C double bond stretching mode of the benzene ring (*ν*_CCr_), respectively.

The *δ*_TCNQ-TCNQ_ modes between the TCNQ anion radical dimer below 820 cm^−1^ were found to disappear approximately 300 fs after photo-excitation. This result implies that the dimer dissociates within this time span.

The *ν*_CCs1.5_ peak at 1425 cm^−1^ just after photo-excitation was separated into blue-shifted and red-shifted bands and transitioned to a new peak at approximately 400 fs. The mode separation between the blue-shifted mode and the red-shifted mode is comparable with the bandwidth of the modes appearing in the spectrogram. The result shown in Appendix D shows that the observed mode branching is not artifact but really exists in the transient reaction. The observed mode branching can be explained by noting that the *ν*_CCs1.5_ mode has a bond order of 1.5. One of the disproportionation products, TCNQ, has a C-C double bond, which generates the blue-shifted band. The other product, TCNQ^2−^, has a C-C single bond that corresponds to the red-shifted band. The TCNQ is generated in the electronic ground state and generates a peak at 1470 cm^−1^, corresponding to the C-C double-bond stretching mode (*ν*_CCs2_). In contrast, the TCNQ^2−^ was in the electronic singlet excited state and produced a band due to the C-C single bond stretching mode (*ν*_CCs1_) at 1340 cm^−1^. This frequency of *ν*_CCs__1_ higher than C-C single bond stretching mode agrees with the theoretical calculation. The blue shift of the frequency is thought to be because the methylene carbon in malononitrile unit has a negative charge of −0.5.

**FIG. 5.**
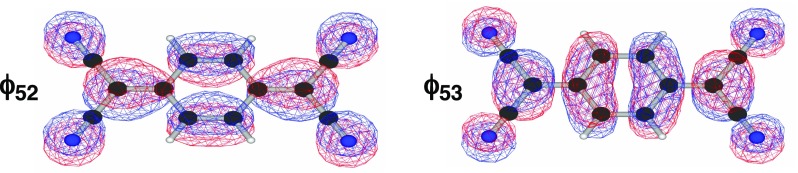
HOMO(*ϕ*_52_) and LUMO(*ϕ*_53_) orbitals of TCNQ calculated using B3LYP/6–31G*.

The changes in bond order can be understood as follows. The HOMO (LUMO) of TCNQ, ϕ_52_ (ϕ_53_), is the bonding (antibonding) orbital of the C-C bond of the side chain group (Fig. 5). Therefore, the bond order of this C-C bond increases in the order of TCNQ > TCNQ^−•^ > TCNQ^2−^ when a single electron is promoted to the ϕ_53_ orbital of TCNQ^−•^ and TCNQ^2−^. The TCNQ^2−^ produced in the singlet excited state makes the transitions to a triplet excited state via inter-system crossing. This results in the gradual blue-shift of the peak from 1340 to 1360 cm^−1^ with a new peak appearing approximately 900 fs after photo-excitation.

The 1560 cm^−1^ peak seen just after photo-excitation broadens 400 fs after photo-excitation. The DFT calculations (Table I) indicated that this peak is associated with frequency shifts of +15 and −5 cm^−1^ for the disproportionation products TCNQ and singlet excited state TCNQ^2−^, respectively. These frequency shifts were observed to occur in ∼400 fs. The *ν*_CCr_ mode of triplet excited state TCNQ^2−^ was predicted to undergo a red-shift to 1550 cm^−1^ and to be Raman inactive. Therefore, this peak should disappear approximately 800 fs after photo-excitation.

The *δ*_C-H_ mode appearing at 1200 cm^−1^ immediately after photo-excitation exhibited a gradual red-shift to 1140 cm^−1^ and is attributed to singlet excited state TCNQ^2–^ present 400 fs after photo-excitation. This same peak separated into blue- and red-shifted bands at 1180 and 985 cm^−1^, respectively, around 900 fs after photo-excitation, as the result of the formation of triplet excited state TCNQ^2−^. These data agree with the results of DFT calculations (Table I).

The *ν*_Bn_ mode observed at 1060 cm^−1^ just after photo-excitation was also split into two peaks, with a gradual frequency shift to 950 cm^−1^ in the case of TCNQ and 1140 cm^−1^ for singlet excited state TCNQ^2−^. This mode was no longer present about 600 fs after photo-excitation because it also becomes Raman inactive due to the formation of triplet excited state TCNQ^2−^, as predicted by DFT calculations (Table I).

The results of the spectrogram analysis demonstrate that the Na^+^_2_[TCNQ^−•^]_2_ dissociated within approximately 400 fs and that the inter-system crossing of TCNQ^2–^ occurred at about 900 fs. These observations are in good agreement with the results of electronic dynamics analysis.^16^ The agreement between the spectrogram and the electronic dynamics was confirmed again using the present sample data as follows.

**FIG. 6.**
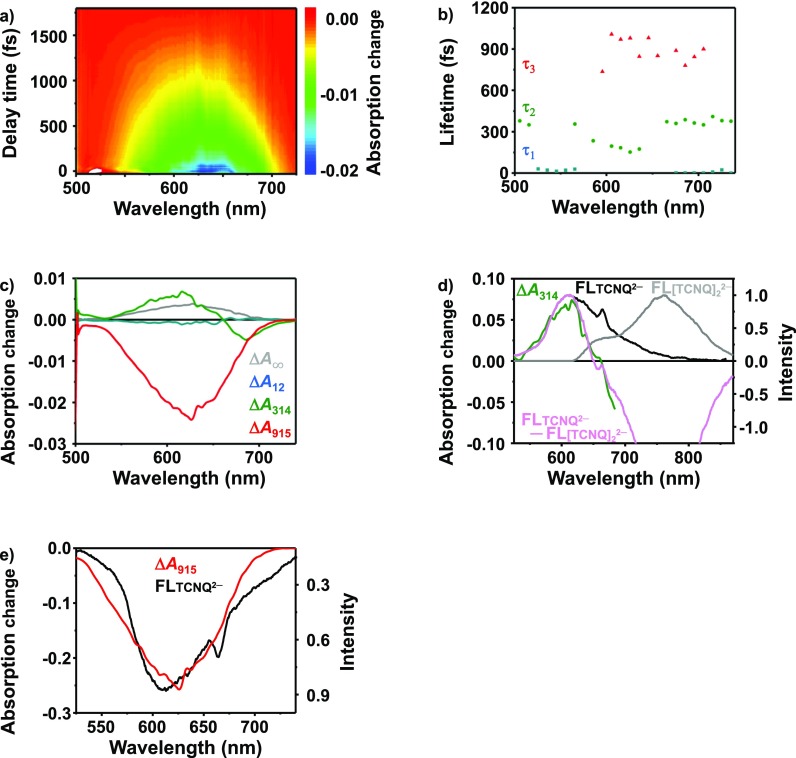
(a) Two dimensional map of the transient absorption signal, (b) lifetimes estimated at each probe wavelength, (c) decay associated spectra (DAS) of corresponding lifetimes, and (d) fluorescence spectra of TCNQ^2−^ and [TCNQ^−•^]_2_, and their difference compared with ΔA_314_. (e) Fluorescence spectrum of TCNQ^2−^ compared with ΔA_915_.

When the fluorescence spectrum of TCNQ^2−^ was subtracted from the fluorescence spectrum of [TCNQ^−•^]_2_, its spectral shape agrees with ΔA_314_ [see Fig. 6(d)]. It indicates that the decay of [TCNQ^−•^]_2_ occurs simultaneously with production of TCNQ^2−^ in ∼300 fs. When TCNQ is produced, the C-C bond of the side chain group changes bond order from 1.5 to 2. Meanwhile, the bond order changes from 1.5 to 1 when TCNQ^2−^ is produced. The obtained time constant of ∼300 fs reflects that TCNQ and TCNQ^2−^ took ∼300 fs to be stabilized into their most stable structure. Then, the vibrational modes corresponding to the ground state of TCNQ and singlet excited state of TCNQ^2–^ have appeared at ∼400 fs on the spectrogram (see Fig. 4). The spectral shape of ΔA_915_ agrees with the fluorescence spectrum of TCNQ^2−^ [see Fig. 6(e)], which implies that the fluorescence lifetime of TCNQ^2–^ is ∼900 fs. It agrees with that the intersystem crossing from the singlet excited state to triplet excited state was observed in ∼900 fs in the spectrogram (see Fig. 4).

This analysis shows that the disproportionation of Na^+^_2_[TCNQ^−•^]_2_ is complete within 350 fs and that the emission lifetime of singlet excited state TCNQ^2−^ is on the order of 900 fs. The lifetime of singlet excited state agrees with that reported for TCNQ derivatives.^21,22^

## CONCLUSIONS

The ultrafast molecular vibrational dynamics of an inter-molecular reaction (the photo-disproportionation of Na^+^_2_[TCNQ^−•^]_2_) were studied. In this compound, two TCNQ^–•^ molecules are bridged by two metal ions, and this structure serves to suppress inter-molecular collisions, which in turn tends to maintain the coherence of molecular vibrations. As such, changes in the molecular vibration frequency during the disproportionation of bimolecule aggregates could be visualized in real time. The data obtained in this manner were found to be in good agreement with the results of DFT calculations. Transient process spectroscopy was previously thought to be solely useful for the observation of intra-molecular reactions. However, the present work has proven that this technique is applicable to many chemical reactions, even those classified as inter-molecular.

## Figures and Tables

**FIG. 7.**
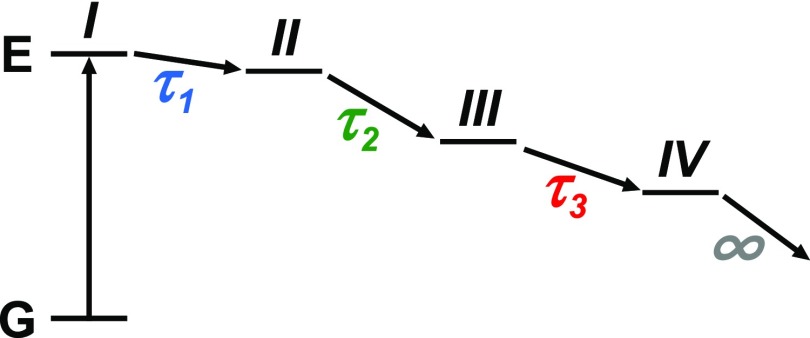
Schematic energy diagram.

**FIG. 8.**
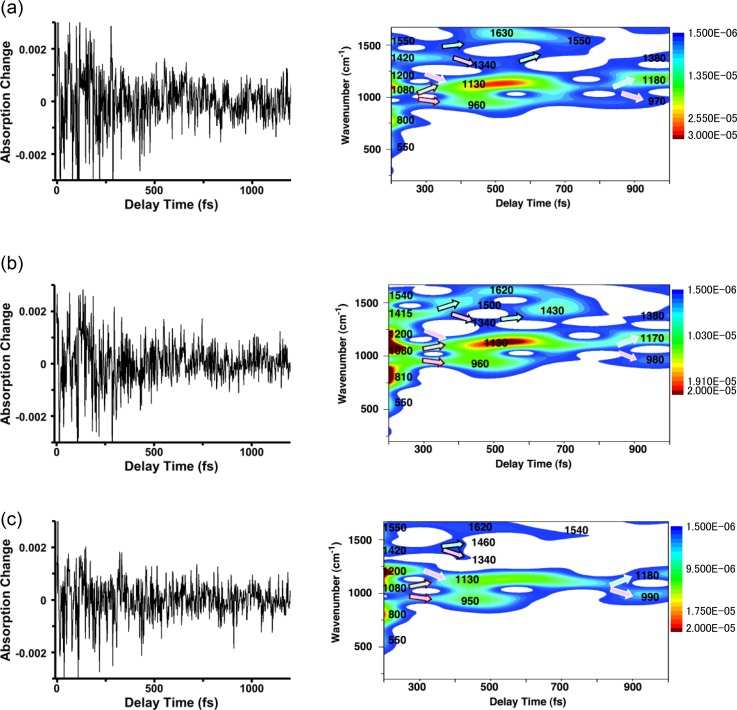
Oscillating components of absorption change traces probed at (a) 699 nm, (b) 701 nm, and (c) 705 nm and their spectrograms.

**FIG. 9.**
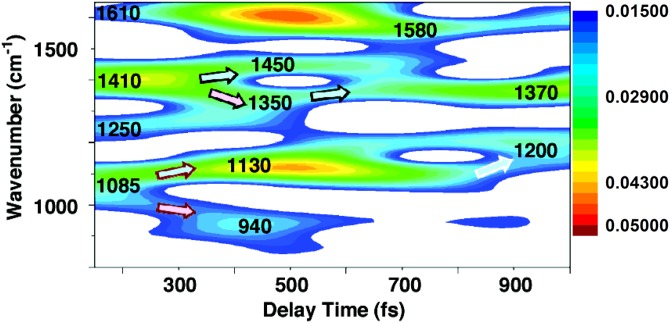
Scalogram calculated from the absorption change trace probed at 700 nm.

**FIG. 10.**
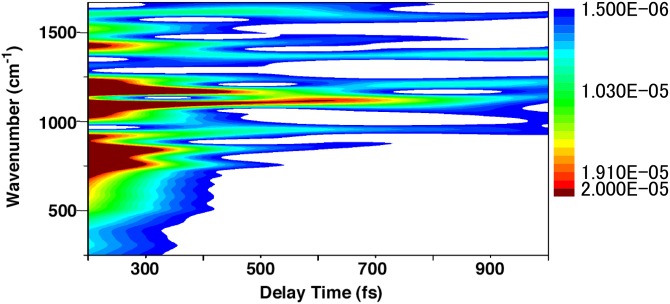
Spectrogram calculated from the absorption change trace probed at 700 nm.

## APPENDIX A: TRANSIENT ABSORPTION

The measured two-dimensional map of transient absorption has shown a negative signal just after photo-excitation [Fig. 6(a)]. The measured transient absorption trace was averaged for neighboring ten probe wavelengths corresponding to 10 nm bandwidth. The averaged trace was fitted by triple exponential function of Eq. (A1) assuming the sequential decay process shown in Fig. 7. The time constants obtained in the fitting are plotted in Fig. 6(b). The time constants were averaged for all of the probe wavelength region which give the three lifetimes of *τ_1_ = *12 fs, *τ_2_* = 314 fs, and *τ_3_* = 915 fs. These three decay lifetimes are used to calculate their decay associated spectrum (DAS) shown in Fig. 6(c). Around the probe wavelength of 700 nm, a positive signal corresponding to stimulated emission was found in the DAS of *τ_2_* and *τ_3_*, which can be assigned to the stimulated emission spectrum of [TCNQ^−•^]_2_ (reactant) and TCNQ^2−^ (product). Figure 6(d) shows that the spectral shape of ΔA_314_ agrees with the difference of the fluorescence spectrum between TCNQ^2−^ and [TCNQ^−•^]_2_. Figure 6(e) shows that the spectral shape of ΔA_915_ agrees with the fluorescence spectrum of TCNQ^2−^. It agrees with our previous work.^16^ Therefore, the measured signal probed at ∼700 nm reflects the reaction dynamics of [TCNQ^−•^]_2_ and TCNQ^2−^, and spectrogram analysis of the transient absorption trace at ∼700 nm elucidates the molecular vibration dynamics during the reaction from the reactant to the product.

$$\begin{array}{c} \Delta A_{\left(t\right)}=\Delta A_{I}e^{-\left(\frac{t}{\tau_{1}}\right)}+\Delta A_{II}\left(e^{-\left(\frac{t}{\tau_{2}}\right)}-e^{-\left(\frac{t}{\tau_{1}}\right)}\right)+\Delta A_{III}\left(e^{-\left(\frac{t}{\tau_{3}}\right)}-e^{-\left(\frac{t}{\tau_{2}}\right)}\right)+\Delta A_{IV}\\ =\left(\Delta A_{I}-\Delta A_{II}\right)e^{-\left(\frac{t}{\tau_{1}}\right)}+\left(\Delta A_{II}-\Delta A_{III}\right)e^{-\left(\frac{t}{\tau_{2}}\right)}+\left(\Delta A_{II}-\Delta A_{III}\right)e^{-\left(\frac{t}{\tau_{2}}\right)}+\left(\Delta A_{III}-\Delta A_{IV}\right)e^{-\left(\frac{t}{\tau_{3}}\right)}+\Delta A_{IV}\\ =\Delta A_{1}e^{-\left(\frac{t}{\tau_{1}}\right)}+\Delta A_{2}e^{-\left(\frac{t}{\tau_{2}}\right)}+\Delta A_{3}e^{-\left(\frac{t}{\tau_{3}}\right)}+\Delta A_{0}.\\ \Delta A_{I}-\Delta A_{II}=\Delta A_{1},\;\Delta A_{\mathrm{II}}-\Delta A_{III}=\Delta A_{2},\;\Delta A_{\mathrm{III}}-\Delta A_{IV}=\Delta A_{3},\;\Delta A_{IV}=\Delta A_{0}.\\ \Leftrightarrow \Delta A_{IV}=\Delta A_{0},\;\Delta A_{\mathrm{III}}=\Delta A_{3}+\Delta A_{0},\;\Delta A_{\mathrm{II}}=\Delta A_{2}+\Delta A_{3}+\Delta A_{0},\Delta A_{I}=\Delta A_{1}+\Delta A_{2}+\Delta A_{3}+\Delta A_{0} \end{array}$$ (A1)

## APPENDIX B: SPECTROGRAM ANALYSIS AT OTHER PROBE WAVELENGTHS

The result of electronic dynamics shows that induced absorption of the reactant, Na^+^_2_[TCNQ^−•^]_2_, appears at around 700 nm. We have studied vibrational dynamics by analyzing the transient absorption trace probed at 700 nm (see Fig. 3). The observed dynamics was confirmed performing the same analysis also at other probe wavelengths as follows.

Figures 8(a) and 8(b) show that spectrograms calculated from the transient absorption trace probed at 699 nm and 701 nm agree with that at 700 nm supporting the reliability of the analyzed result. The spectrogram calculated from the transient absorption trace probed at 705 nm [Fig. 8(c)] also shows the same result even though high frequency modes disappear in the middle of the reaction because their intensities are lower than that at ∼700 nm. At probe wavelength longer than 705 nm, spectrogram analysis could not be performed because of the low signal-to-noise ratio.

## APPENDIX C: SCALOGRAM ANALYSIS

The vibrational dynamics observed by the spectrogram analysis was also confirmed by performing scalogram analysis. The scalogram analysis was performed for the absorption change trace probed at 700 nm using a complex Morlet wavelet, and the result is plotted in Fig. 9.

The result of the scalogram analysis shown in Fig. 9 shows good agreement with the molecular vibration dynamics observed in Fig. 4 analyzed by the spectrogram analysis. It confirmed that the observed shift of molecular vibration frequencies reflects the molecular vibration dynamics not being caused by artifacts in those analysis methods.

## APPENDIX D: SPECTROGRAM ANALYSIS WITH LONGER TIME GATE WIDTH

The *ν*_CCs1.5_ peak at 1425 cm^−1^ just after photo-excitation was separated into blue-shifted and red-shifted bands and transitioned to a new peak at approximately 400 fs. The mode separation between the blue-shifted mode and the red-shifted mode is comparable with the bandwidth of the modes appearing in the spectrogram. Therefore, it is necessary to confirm whether the mode branching really exist or is artifact from poor resolution. As shown in Fig. 10, we have calculated another spectrogram using broader temporal gate width of 500 fs which corresponds to the spectral resolution of ∼70 cm^−1^.

These spectrograms shown in Fig. 10 show the same mode separation between the blue-shifted mode and the red-shifted mode, which indicates that the observed mode branching is not artifact but really exists in the transient reaction.
